# Prevalence and inter-relationship of different Doppler measures of dyssynchrony in patients with heart failure and prolonged QRS: a report from CARE-HF

**DOI:** 10.1186/1476-7120-7-1

**Published:** 2009-01-07

**Authors:** Magnus Edner, Yong Kim, Knud Norregaard Hansen, Henrik Nissen, Geert Espersen, Karl La Rosee, Fikru Maru, Nick Freemantle, John Cleland, Peter Sogaard

**Affiliations:** 1 Karolinska Institutet, Department of Clinical Sciences, Danderyd Hospital, Stockholm, Sweden; 2 Dept. of Cardiology, Skejby Hospital, Aarhus University Hospital, Denmark; 3 Dept of Cardiology, Odense C Hospital, Denmark; 4 University of Cologne, Cologne, Germany; 5 The University of Birmingham, Edgbaston, UK; 6 Dept of Cardiology, Castle Hill Hospital, Kingston-upon-Hull, UK; 7 Dept of Cardiology, Gentofte University Hospital, Denmark

## Abstract

**Background:**

Cardiac resynchronisation therapy (CRT) improves mortality and morbidity in heart failure patients with wide QRS. Observational studies suggest that patients having more left ventricular dyssynchrony pre-implantation obtain greater benefit on ventricular function and symptoms with CRT.

**Aim:**

To provide an analysis of the prevalence and type of dyssynchrony in patients included in the CARE-HF trial.

**Methods:**

100 patients 67 (58 to 71) years were examined with echocardiography including tissue doppler imaging before receiving a CRT-pacemaker. Atrio-ventricular dyssynchrony (LVFT/RR) was defined as left ventricular filling time <40% of the RR-interval. Inter-ventricular mechanical delay (IVMD) was measured as the difference in onset of Doppler-flow in the pulmonary and aortic outflow tracts >40 ms. Intra-ventricular (regional) dyssynchrony in a 16-segment model was expressed either as a delayed longitudinal contraction (DLC) during the postsystolic phase or by tissue synchronisation imaging (TSI) with a predefined time-difference in systolic maximal velocities >85 ms.

**Results:**

LVFT/RR was present in 34% and IVMD in 60% of patients while intra-ventricular dyssynchrony was present in 85% (DLC) and 86% (TSI) with a high agreement between the measures (Kappascore 0.86–1.00), indicating the methods being interchangeable. Patients with cardiomyopathy (53%) were more likely to have LVFT/RR <40% (45% vs. 21% (p= 0.02)) and more segments affected by intra-ventricular dyssynchrony 4(3, 5) vs. 3(1, 4), p = 0.002, compared to patients with ischemic heart disease.

**Conclusion:**

The prevalence of intra-ventricular dyssynchrony is high in patients with heart failure, wide QRS and depressed systolic function. Most important, TSI appears to be a fast and reliable method to identify patients with intra-ventricular dyssynchrony likely to benefit from CRT.

## Background

Heart failure (HF) is common and despite recent advances in pharmacological therapy often debilitating [1-4]. Increased QRS duration on the surface electrocardiogram, reflecting regional delays in electrical activation, is present in more than one third of HF patients and has been associated with worse prognosis independent of the level of left ventricular (LV) ejection fraction (EF), rhythm and age [5]. Regional delays in electrical activation may cause regional delays in contraction and relaxation resulting in intra-ventricular dyssynchrony and a decline in ventricular function and efficiency [6]. However, QRS duration is probably only an approximate guide to the presence of dyssynchrony, Dyssynchrony can be classified as atrio-ventricular, inter-ventricular and intra-ventricular. There is some evidence that intra- but not inter-ventricular dyssynchrony predicts deaths or worsening heart failure in patients with idiopathic dilated cardiomyopathy (IDCM) [7,8]. Assessment of cardiac dyssynchrony by imaging may identify the magnitude of the response to cardiac resynchronization therapy (CRT), in terms of improved cardiac function, symptoms and possibly prognosis, although data from relatively small observational studies must be interpreted with caution [9-12].

The aim of this study is to describe the prevalence of atrio-ventricular, inter-ventricular and intra-ventricular dyssynchrony by different Doppler techniques in patients with and without ischemic heart disease in a subpopulation of the CARE-HF trial.

## Methods

### Patients

The CARE-HF trial enrolled patients with New York Heart Association (NYHA) class III or IV despite pharmacological treatment, with a QRS duration ≥120 ms, LV ejection fraction (EF) ≤35%, LV end diastolic diameter ≥30 mm/m^2 ^(height in m) and in sinus rhythm. Patients with a QRS duration between 120 ms and 149 ms required at least two of the following dyssynchrony criteria: (1) aortic pre-ejection delay >140 ms; (2) interventricular mechanical delay >40 ms; and (3) delayed activation of the posterolateral wall of the left ventricle as shown by any overlap between time from QRS to peak systolic movement of the posterior wall at m-mode and time from QRS to the beginning of the E-wave at pulsed Doppler transmitral flow. Patients with a QRS duration >150 ms did not require echo confirmation of dyssynchrony.

Of the 813 patients enrolled in the trial, 735 had an analysable echocardiographic examination. [13] including 92 patients with a QRS duration of between 120 ms and 149 ms. One-hundred patients aged 67.1 ± 10,4 years and randomized to the CARE-HF trial in five TDI interested and geographically close participating centers in Denmark, Germany and Sweden were eligible to the current sub study. The study was approved by the local ethics committees and all patients gave written informed consent.

### Tissue Doppler echocardiography

**Figure 1:**
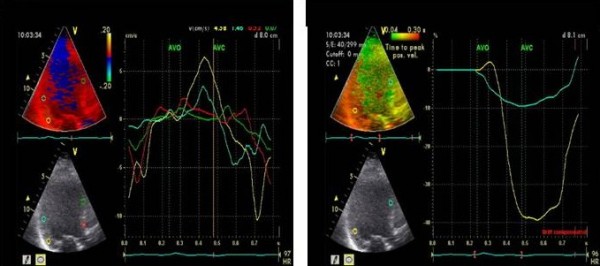
**TVI-, DLC-curves and TSI illustrating dyssynchrony**. TVI curves (left) showing late systolic peak velocity in the posterior segments (yellow and blue). TSI (right), is a signal-processing algorithm of the tissue Doppler data to automatically detect peak positive velocity and then colour-code the time to peak velocities in green for normal timing, yellow-orange for moderate delay, and red for severe delay, in peak velocity showing dyssynchrony (orange) in the corresponding posterior segments and SR curves showing delayed longitudinal contraction (DLC, yellow) in the same segment after aortic valve closure (AVC).

Patients were examined in the left lateral position and tissue Doppler echocardiography was acquired in the apical 4 -chamber, 2-chamber and long-axis views using a VIVID-5 system with a 2,5 MHz transducer (GE-Vingmed Ultrasound, Horten, Norway). An average of 2–4 RR-intervals were stored as a cine loop (from p-wave to p-wave) on an optical disc for later off-line analysis (Echo-Pac Software, GE-Vingmed Ultrasound, Horten, Norway). Systole was defined as the ejection time from opening until closure of the aortic valves as measured by tissue velocity imaging (TVI) in the apical long-axis view [14]. The time from onset of the QRS-complex until opening of the aortic and pulmonary valves were defined as the pre-ejection periods. From the TVI loops colour Doppler TVI analysis was performed in each of the apical views. The 16 segment LV-model of the American Society of Echocardiography was used for regional analysis of the extent of dyssynchrony [15]. The extent of dyssynchrony during systole was evaluated regionally by tissue synchronisation imaging (TSI) (Figure 1). The TSI-start was set to the aortic valve opening and the TSI-end to the aortic valve closure and the TSI cut-off value was preset to 85 ms. This cut-off value, assumed to indicate important intra-ventricular dyssynchrony, was chosen based on the application of the method in previous patients in our echo-laboratory, and now tested in a prospective trial.

Presence or absence of delayed longitudinal contraction (DLC), i.e. motion towards the apex following closure of the aortic valve was recorded by strain rate (SR) technique in each of the 16 LV segments [16]. Motion toward the apex after closure of the aortic valve was only registered as DLC if negative SR documented that the motion reflected true shortening (Figure 1). All the TVI analyses were performed twice by the same investigator and the mean of the two measurements was used. Intra- and inter-variability was fairly low. The coefficient of variation for TSI and DLC analysis was 3 and 5%, and 4,5 and 9%, respectively.

### Atrio-ventricular and Inter-ventricular dyssynchrony

Atrio-ventricular dyssynchrony (LVFT/RR) was considered present if the left ventricular filling time (LVFT) measured from the mitral valve Doppler in-flow signal was <40% of the cardiac interval (R-R). The inter-ventricular mechanical delay (IVMD), was defined as the time difference between the onset of forward flow in the LV outflow tract and RV outflow tract and >40 ms was considered indicative IVMD [17]

### Other methods

Other aspects of the study methods such as assays for NT-proBNP have been published in detail [17].

#### Statistical analysis

Descriptive statistics were used to analyse the study population and median (interquartile range) are given unless otherwise stated. Difference in proportions was assessed using Fisher's exact test, and the Kruskal Wallis test was used to compare non normally distributed continuous data. Different variables all known to be risk-factors in the heart failure syndrome were tested by univariate analysis for relations with intra-ventricular dyssynchrony measured by TSI and DLC separately. All tested risk-factors with a p-value < 0.10 were also tested in a multiple regression analyses in order to find out which variables that might be independently most important for intra-ventricular dyssynchrony. Tested variables were examined through developing generalised linear models, with log link functions and Poisson error structure. Thus, the statistical analysis attempts to predict the presence and severity of dyssynchrony in a subject. Multivariate models used forward stepwise selection, with a P value of 0.10 for entry to the model, and p = 0.05 to stay in the model. All analyses were conducted using SAS 9.1 (SAS Institute, Cary, NC).

## Results

### Baseline characteristics of the patients

Eight hundred and thirteen patients were recruited to the CARE-HF trial of which 100 participated in the tissue Doppler sub study. Only nine patients in the sub study had QRS below 150 ms. The characteristics of the patients in the sub study were similar to those in the main-study, except for evidence for more intense pharmacological management (Table 1 and Table 2). Echocardiographic measures of dimensions and LV EF were however similar and there was only a trend to higher (N.S.) NT-proBNP.

**Table 1:** Baseline Characteristics

**Variable, n (%)**	**Sub study Patients** **(N = 100)**	**Non Sub study Patients** **(N = 713)**	**P value**
Age [years]	67.1 (57.6, 71.3)	66.4 (59.5, 72.0)	0.71
Male gender	66 (66.0)	531 (74.5)	0.09
Ischemic History	47 (47.0)	292 (41.0)	0.28
Diabetes	24 (24.0)	183 (25.7)	0.81
Duration of HF, [months]	4.3 (1.5, 8.1)	4.0 (1.5, 7.9)	0.80
ACE/ARB	97 (97.0)	673 (94.4)	0.35
Betablockers	83 (83.0)	503 (70.6)	0.009
Loop Diuretics ≥ 80 mg furosemide or equivalent	63 (63.0)	289 (40.5)	<.0001
Aldosterone antagonists	73 (73.0)	384 (53.9)	0.0003
Digoxin	53 (53.0)	293 (41.1)	0.03
Aspirin	68 (68.0)	292 (41.0)	<.0001
Warfarin	28 (28.0)	249 (34.9)	0.18
Statins	37 (37.0)	284 (39.8)	0.66

HF = heart failure, ACE = angiotensin converting enzyme inhibitors, ARB = angiotensin receptor blockers. Median, (IQR).

**Table 2:** ECG, NT-proBNP and echocardiography.

	**Sub study patients** **(N = 100)**	**Non Sub study patients** **(N = 713)**	**P value**
Heart rate [bpm]	69 (60, 78)	69 (61, 78)	0.93
QRS [ms]	160 (150, 172)	160 (152, 180)	0.20
Systolic blood pressure, (mm Hg)	120 (108, 130)	117 (105, 130)	0.76
Diastolic blood pressure, (mm Hg)	70 (64, 80)	70 (60, 80)	0.21
N-terminal-pro-brain natriuretic peptide (pg/ml)	2275 (905, 5277)	1768 (736, 4009)	0.06
Enddiastolic diameter (mm)	7.5 (6.5, 8.0)	7.1 (6.4, 7.8)	0.87
Endsystolic diameter [mm]	6.1 (5.4, 6.9)	6.4 (5.5, 7.1)	0.79
Ejection fraktion, [%]	24.6 (20.4, 30.2)	24.7 (21.7, 28.9)	0.94

Median (IQR), ECG = electrocardiogram.

### Atrio-ventricular and Inter-ventricular dyssynchrony

Thirty-four patients (34%) had a LVFT/RR <40%, 60 (60%) had inter-ventricular dyssynchrony and the median IVMD was 47.9 ms (Table 3).

**Table 3:** Signs of dyssynchrony in the sub study patients, n = 100

**Variable**	**All pts** **(N = 100)**	**Pts with IDCM** **(N = 53)**	**Pts with IHD** **(N = 47)**	**P value** **CMP vs IHD**
LVFT, ms,	400.0 (312.6, 494.9)	372.8 (280.8, 477.1)	426.9 (346.5, 519.1)	0.03
LVFT as % of RR	44.6 (38.6, 52.1)	42.5 (36.4, 50.2)	47.0 (41.5, 54.1)	0.02
No of pts <40% (%)	34 (34.0)	24 (45.3)	10 (21.3)	0.02
IVMD, ms	47.9 (27.4, 62.0)	50.5 (33.3, 69.7)	41.1(23.9, 58.3)	0.11
No of pts with IVMD >40 ms (%)	60 (60.0)	37 (69.8)	23 (48.9)	0.09
Base-segments [6]				
No of pts with DLC(%)	85 (85.0)	47 (88.7)	38 (80.9)	0.40
No of segments with DLC	2 (1, 3)	3 (2, 3)	2 (1, 2)	0.05
Mid-segments [6]				
No of pts with DLC(%)	70 (70.0)	42 (79.2)	28 (59.6)	0.05
No of segments with DLC	2 (0, 2)	2 (1, 2)	1 (0, 2)	0.02
Apical-segments, [4]				
No of pts with DLC	0	0	0	
16-segments model				
No of pts with DLC	85 (85.0)	47 (88.7)	38 (80.9)	0.40
No of segments with DLC	4 (2, 5)	4 (3, 5)	3 (1, 4)	0.003
Base-segments [6]				
No of pts with TSI(%)	86 (86.0)	48 (90.6)	38 (80.9)	0.25
No of segments with TSI	2 (1, 3)	3 (2, 3)	2 (1, 2)	0.03
Mid-segments [6]				
No of pts with TSI(%)	69 (69.0)	42 (79.2)	'27 (57.4)	0.03
No of segments with TSI	2 (0, 2)	2 (1, 2)	1 (0, 2)	0.03
Apical segments, 4				
No of pts with TSI	0	0	0	
16-segments model				
No of pts with TSI(%)	86 (86.0)	48 (90.6)	38 (80.9)	0.25
No of segments with TSI	4 (2, 5)	4 (3, 5)	3 (1, 4)	0.002

Median (IQR), n (%). LVFT = left ventricular filling time, IVMD = Inter ventricular mechanical delay, DLC = delayed longitudinal contraction, TSI = tissue synchronisation imaging, IDCM = idiopathic dilated cardiomyopathy, IHD = ischemic heart disease.

### Intra-ventricular dyssynchrony

**Figure 2:**
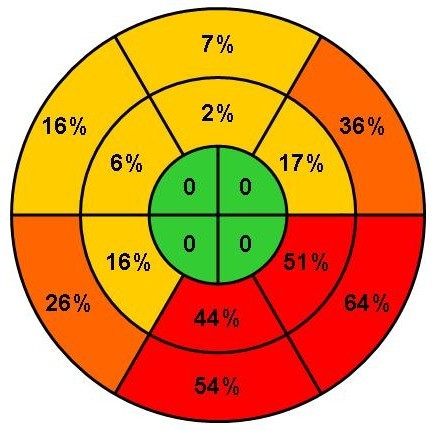
**Distribution of intra-ventricular dyssynchrony in affected segments in the heart failure population, n = 100**.

Eighty-six patients (86%) had intra-ventricular dyssynchrony on TSI. The distribution of TSI-measured dyssynchrony is shown in figure 2. The median number of affected segments was four and all showed dyssynchrony in one or more basal segment. Mid-segments were affected in 69 patients, with a median of two affected segments, but none had apical dyssynchrony. Eighty-five patients (85%) had intra-ventricular dyssynchrony measured by the SR technique, all 85 patients having affected basal segments, with a median two out of six segments, 70 having mid-segments affected but none having apical dyssynchrony (Table 3).

On univariate analysis, patients with dyssynchrony measured by TSI were more likely to be women p = 0.001, have IDCM p = 0.002 and have greater QRS duration p = 0.03. Findings were similar for intra-ventricular dyssynchrony by DLC (Table 4).

**Table 4:** TSI and DLC count univariate analysis

	**TSI**		**DLC**	
Variable	Relative Risk (95 CI)	P value	Relative Risk (95% CI)	P value

Age	0.998 (0.988 to 1.007)	0.625	0.997 (0.987 to 1.007)	0.52
Male gender	0.698 (0.563 to 0.866)	0.001	0.692 (0.559 to 0.858)	0.0007
IHD	0.710 (0.563 to 0.866)	0.002	0.718 (0.576 to 0.890)	0.003
Heart rate	1.008 (0.999 to 1.016)	0.068	1.008 (0.999 to 1.016)	0.08
Systolic BP, mmHg	1.003 (0.997 to 1.009)	0.313	1.003 (0.997 to 1.009)	0.26
NT-proBNP, pg/ml	0.984 (0.909 to 1.065)	0.685	0.982 (0.908 to 1.062)	0.65
QRS, ms	1.006 (1.001 to 1.012)	0.032	1.006 (1.000 to 1.012)	0.03
LVESVi, ml/m^2^	1.002 (1.000 to 1.004)	0.0694	1.002 (1.000 to 1.004)	0.08
LVEDVi, ml/m^2^	1.002 (1.000 to 1.004)	0.0984	1.002 (1.000 to 1.004)	0.12
EF, %	0.987 (0.970 to 1.003)	0.11	0.986 (0.970 to 1.002)	0.10

IHD = ischemic heart disease, BP = blood pressure, NT-proBNP = N-terminal brain natriuretic peptide, LVESVi = left ventricular end systolic volume/m^2^, EF = ejection fraction.

On multi-variate analysis woman, patients with IDCM and measures of ventricular dilatation and dysfunction, but not QRS duration, predicted the presence of dyssynchrony by TSI and DLC.

### Inter-relationship between TSI and DLC

Most patients had intra-ventricular dyssynchrony using both methods but measurements of TSI are substantially faster. Accordingly, we investigated the agreement between the methods analysing every segment separately in the 16-segment model [15] to determine wheather their results were interchangeable. Within the six basal segments, three had a perfect match and the Kappa coefficient was 0.86–1.0. The agreement within the mid-segments was even better with three segments showing perfect match and the Kappa coefficient being 0.92–1.0. These findings suggests that TSI and DLC are interchangeable methods with which to assess dyssynchrony.

### Presence of different types of dyssynchrony in patients with and without intra-ventricular dyssynchrony

LVFT/RR <40% was present in 33% and IVMD in 51% of patients with intra-ventricular dyssynchrony (TSI or DLC). Conversely, 85% of the patients with IVMD >40 ms had intra-ventricular dyssynchrony.

All three types of dyssynchrony co-existed in only 23% of the patients (Table 5).

**Table 5:** Combinations of different types of dyssynchrony, n = 100

**Variable**	**All pts** **N = 100**	**Pts with IDCM** **N = 53**	**Pts with IHD** **N = 47**	**CMP vs. IHD** **P value**
LVFT/RR + IVMD, (%)	25 (25.0)	19 (35.8)	6 (12.7)	0.02
LVFT/RR + Intra-v (DLC), (%)	32 (32.0)	23 (43.4)	9 (19.1)	0.02
LVFT/RR + Intra-v (TSI), (%)	33 (33.0)	24 (45.3)	9 (19.1)	0.01
LVFT//RR + Intra-v (DLC or TSI), (%)	33 (33.0)	24 (45.3)	9 (19.1)	0.02
IVMD + Intra-v, (DLC or TSI) (%)	51 (51.0)	33 (62.3)	18 (38.3)	0.04
LVFT/RR + IVMD + Intra-v (DLC or TSI), (%)	23 (23.0)	18 (34.0)	5 (10.6)	0.01

Abbreviations see table 3.

### The impact of aetiology – cardiomyopathy or ischemic heart disease on LV dyssynchrony

**Figure 3:**
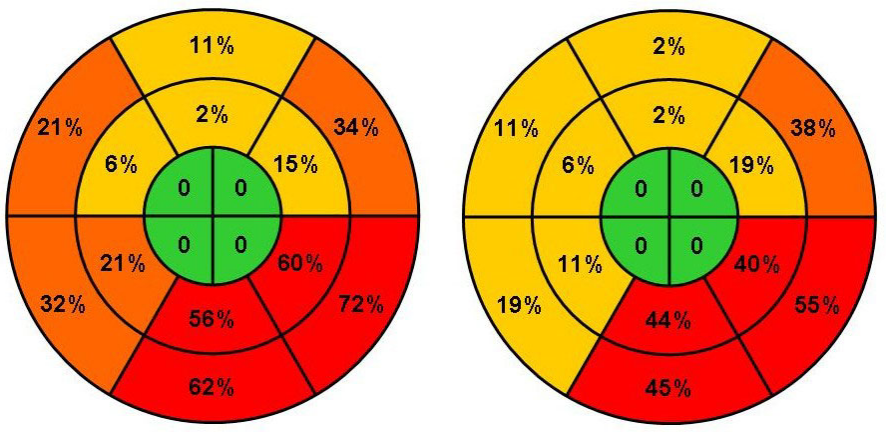
**Distribution of intra-ventricular dyssynchrony in affected segments in patients with cardiomyopathy n = 53 (left) and ischemic heart disease n = 47 (right)**.

Patients with cardiomyopathy had shorter LVFT and more LVFT/RR than those with ischemic heart disease, 45,3% vs. 21,3%, p = 0.02. IVMD was present in 69% of patients with IDCM and 48,9% of those with IHD, p = 0.09. Similar numbers of patients with IDCM and IHD had intra-ventricular dyssynchrony, with the basal lateral and posterior segments most commonly affected (Figure 3), but patients with IDCM had more affected segments, with a median of 4(3, 5) vs. 3(1, 4), p = 0.027 (Table 3 and 5).

## Discussion

In this cohort of 100 patients with moderate to severe heart failure, reduced systolic LV function and prolonged QRS duration we found a high prevalence and severity of each type of dyssynchrony. Intra-ventricular dyssynchrony was present in the great majority of patients measured by either tissue Doppler imaging method both of which appear to identify patients who respond better to CRT in observational trials. Reports of the prevalence of intra-ventricular dyssynchrony vary depending on the method used. Previous reports have suggested a prevalence of 72–80% in patients with heart failure and QRS duration >120 ms [18-20] which is similar to the rate reported in his study.

IVMD, one of the potential entry criteria for CARE-HF tended to be less prevalent but occurred in 60% of the patients in this sub study, a similar proportion (62%) to that observed in the overall study [10] and in previous reports [18,19]. The rates they report are entirely consistent with these data. Intra-ventricular dyssynchrony was equally common in patients with and without IVMD. It would appear that inter- and intra-ventricular dyssynchrony are both common but one does not appear to influence the prevalence of the other. Why are IVMD and intra-ventricular dyssynchrony not linked (0.86 multiplied by 0.6 = 0.51 i.e. 51% is a simple proportion which it should not be if they were linked). This is somewhat surprising since greater intra-ventricular dyssynchrony should allow the intra-ventricular septum to contract earlier leading to a shorter pre-ejection period and impaired LV function leading to longer aortic pre-ejection time. However, the most likely explanation to this is that IVMD reflects a global dyssynchrony but intra-ventricular dyssynchrony a more heterogeneous regional dyssynchrony as described in figure 2. The agreement between inter-and intra -ventricular dyssynchrony has been reported low [18].

### Inter-ventricular and intra-ventricular dyssynchrony

Many studies show that QRS duration predicts mortality in patients with heart failure. It is assumed by many that this reflects more severe dyssynchrony. However, QRS also reflects LVEF [21]. There are few data that show whether dyssynchrony by imaging is prognostically important. In the CARE-HF study, IVMD was associated with a better prognosis in the control group. One small observational study [8] with an unusually high mortality found that intra -ventricular dyssynchrony but not IVMD or QRS predicted mortality.

The QRS width has been shown to be related to both inter- and intra-ventricular dyssynchrony but during long-term follow up of patients with dilated cardiomyopathy only intra-ventricular dyssynchrony was related to cardiac events [7,8]. Another important issue is the capability to predict LV reverse remodelling after CRT and even if IVMD has been shown to predict response to CRT [22] most investigators consider intra-ventricular dyssynchrony to select responders to CRT [23,24]. On the other hand, IVMD is easy to measure and in this study 85% of the patients with IVMD >40 ms also had intra-ventricular dyssynchrony which could make it reasonable to include IVMD in clinical routine.

### Tissue synchronisation imaging and strain rate

In this study, we found a high prevalence of intra-ventricular dyssynchrony with a high agreement between the two methods suggesting that they are inter-changeable in terms of evaluating intra-ventricular dyssynchrony. Thus a postsystolic DLC is associated with a delayed maximal velocity in the same region during systole.

**Figure 4:**
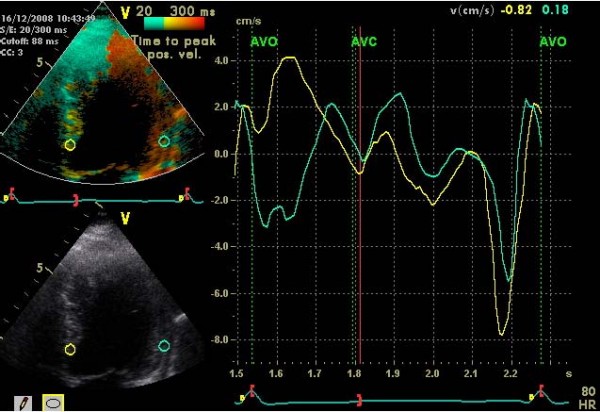
**Tissue synchronization imaging (TSI) showing delayed lateral systolic contraction in red colour with corresponding TVI-curves**. AVO and AVC = aortic valve opening and closure.

**Figure 5:**
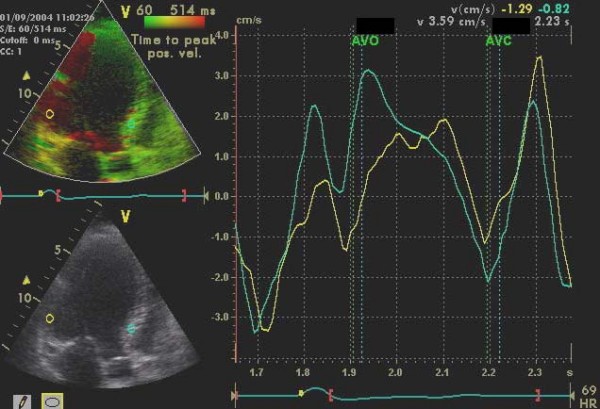
**Tissue synchronization imaging (TSI) showing inferior delayed systolic contraction in red colour with corresponding TVI-curves**. The TSI-technique is timesaving and makes it easy to find intra ventricular regions with dyssynchrony. Abbreviations see fig 4.

Since the TSI analyse is substantially faster to perform, TSI may be used in clinical routine. In a previous report by Gorscan et al a cut-off value of >65 ms had a sensitivity and specificity of 87% and 100%, respectively, to predict the initial response to CRT [25]. LV reverse remodelling after three months of CRT may also be predicted by TSI with a sensitivity of 87% and specificity of 81% [26]. Van de Veire et al found there was an excellent correlation between LV dyssynchrony measured manually by color-coded TDI and automatically using TSI [27]. The recently published PROSPECT trial, an observational study, tested twelve different techniques to describe dyssynchrony and many of those turned out to be difficult, due to high variability, to use in a multicenter study [28]. As a mater of fact, those techniques which could be said are easiest to use i.e. lateral- septum maximal velocity difference (T_S _-lateral- septum), LVFT/RR, APE and IVMD turned out to be the most useful. This further support the use of the fast and simple TSI technique (Fig 4, 5). In our preliminary analysis of outcome data at least two TSI positive segments are needed to predict a responder to CRT.

However, analysis of CARE-HF suggests that the initial response of LV function to CRT is a poor predictor of the effect of intervention on long-term mortality. Strain rate analyses of delayed longitudinal contraction (DLC) often require an off-line approach and are therefore more time-consuming. The main advantage with strain rate is its ability to differentiate between active systolic contraction and passive motion. The number of segments with DLC is related to the improvement in LV systolic function by biventricular pacing during long-term follow-up [11]. Thus, both methods might be useful to select patients who get greater benefit from CRT. However, randomized controlled trials (RCTs) that might be difficult to design will be required to test this hypothesis.

### Cardiomyopathy or ischemic heart disease

We found patients with cardiomyopathy to have shorter LVFT than those with ischemic ethiology and more patients had LVFT/RR <40% of the R-R interval. There was a tendency to more patients having intra-ventricular dyssynchrony in the CMP group compared to IHD, and there was a difference in the number of affected segments 4(3, 5) vs. 3(1, 4), p = 0.027. Thus, two types of dyssynchrony and combinations of dyssynchrony were more common in patients with dilated cardiomyopathy. Similar findings with signs of less dyssynchrony in patients with ischemic heart failure were shown in a study by Zwanenburg et al using Magnetic resonance imaging [29]. Thus, CRT might be less effective in ischemic patients compared with patients with cardiomyopathy (but CARE-HF shows ischemic patients to have smaller effect on LV function but similar effect on long-term mortality). This has been observed in i.e. the MIRACLE trial in which both non-ischemic and ischemic patients improved regarding reverse remodelling and enhanced EF but the changes was statistically significantly smaller in the ischemic group [30]. This difference has also been observed in other studies [31,32] and could thus be explained by less dyssynchrony in the ischemic patients but perhaps also by the ischemic patients having more scar tissue.

### Limitations of the study

The PROSPECT (Predictors of response to cardiac resynchronization therapy) study was recently reported at the hotline session during the ESC meeting 2007 and commented on by E. S Zegers and F.W.A Verheugt in the Euro Heart J [33]. This is a multicentre study including 426 patients with standard indication for CRT. The aim was to identify echocardiographic and tissue Doppler measures of dyssynchrony and their ability to predict response to CRT. It was found that presence of signs of dyssynchrony was linked to 11–13% additional clinical response to CRT and 13–23% additional response for reverse remodelling compared to absence of measures of dyssynchrony. One limitation of our study is we do not report on outcome of the patients, however, that was not the aim of this study but obviously it is important to identify signs of dyssynchrony. Another finding in the PROSPECT study was a high inter-variability measuring signs of dyssynchrony and therefore a need for simplification of methodology. However, it is important to remember this was not a RCT but an observational study which means that the importance of dyssynchrony can not be fully estimated. TSI might be such a simple method as described in this paper. It is also easy to measure LVFT/RR and IVMD and using these three signs of dyssynchrony in clinical routine might be sufficient to identify potential responders to CRT.

#### Other examinations

It appears important to assess the amount of scars, especially if the patient has IHD, and LV cardiac reserve which can be done by i.e. magnetic resonance imaging (MRI) or dobutamine stress echocardiography (DSE). Da Costa et al found a 25% increase of LVEF during low-dose DSE and IVMD>50 ms to have independent predictive value to predict response to CRT [34]. There is also an on-going multicenter study, LODO-CRT, testing the value of low-dose DSE before implanting biventricular pacemaker in heart failure patients with standard indication for CRT [35]. Other potentially valuable examinations might be i.e. 3D- echocardiography and speckle tracking echocardiography.

## Conclusion

The prevalence of intra-ventricular dyssynchrony is high in patients with heart failure, wide QRS and severely depressed systolic function. Patients with cardiomyopathy were more likely to have dyssynchrony compared to ischemic patients. We recommend using TSI to identify patients with intra-ventricular dyssynchrony since TSI is an easy method to use and appears to be a reliable and time-efficient method to identify patients likely to benefit from biventricular pacing.

## Competing interests

The authors declare that they have no competing interests.

## Authors' contributions

ME: Made substantial contributions to conception and design, acquisition, analysis and interpretation of data, mainly responsible for drafting the manuscript. YK: acquisition, analysis and interpretation of data and drafting the manuscript. KNK: acquisition, analysis and interpretation of data. GE: acquisition, analysis and interpretation of data. KLR: acquisition, analysis and interpretation of data. FM: acquisition, analysis and interpretation of data. NF: statistical analysis and interpretation of data. JGFC: analysis and interpretation of data, drafting the manuscript. PS: acquisition, analysis and interpretation of data, drafting the manuscript. All authors approved the final version.
